# Evaluation of the owner's perception in the use of homemade diets for the nutritional management of dogs*

**DOI:** 10.1017/jns.2014.24

**Published:** 2014-09-25

**Authors:** Michele C. C. Oliveira, Márcio A. Brunetto, Flávio L. da Silva, Juliana T. Jeremias, Letícia Tortola, Marcia O. S. Gomes, Aulus C. Carciofi

**Affiliations:** 1 College of Agrarian and Veterinarian Sciences (FCAV), São Paulo State University (UNESP), Via de Acesso Professor Paulo Donato Castellane, s/n Jaboticabal 14.884-900, SP, Brazil; 2 Faculty of Veterinary Medicine and Animal Science (FMVZ), University of São Paulo (USP), Av. Duque de Caxias Norte, 225, Pirassununga 13.635-900, SP, Brazil; 3 Technical Department, Mogiana Alimentos (Guabi), Rua das Magnólias, 2405, Campinas 13.050-89, SP, Brazil; 4 Camilo Castelo Branco University (UNICASTELO), Av. Hilário da Silva Passos, 950, Descalvado 13.690-000, SP, Brazil

**Keywords:** Unconventional diets, Owned dogs, Canine nutrition

## Abstract

Many dog owners see homemade diets as a way of increasing the bond with their pets, even though they may not have the convenience of commercial diets. Modifications of ingredients, quality and proportion might change the nutritional composition of the diet, generating nutritional imbalances. The present study evaluated how dog owners use and adhere to homemade diets prescribed by veterinary nutritionists over an extended period of time. Forty-six owners of dogs fed a homemade diet for at least 6 months were selected for the present study. Owners were invited to answer questions by first reading all possible answers and then selecting the one that best indicated their opinion. The results were evaluated through descriptive statistics. Thirty-five owners (76·1 %) found that the diets are easy to prepare. Fourteen owners (30·4 %) admitted to modifying the diets, 40 % did not adequately control the amount of provided ingredients, 73·9 % did not use the recommended amounts of soyabean oil and salt, and 34·8 % did not correctly use the vitamin, mineral or amino acid supplements. Twenty-six owners (56·5 %) reported that their dogs refused to eat at least one food item. All of these alterations make the nutritional composition of the diets unpredictable and likely nutritionally imbalanced. Although homemade diets could be a useful tool for the nutritional management of dogs with certain diseases, not all owners are able to appropriately use this type of diet and adhere to it for an extended period of time and this limitation needs to be considered when recommending the use of homemade diets.

Although the majority of pet owners prefer nutritionally complete and balanced commercial diets, some are interested in providing homemade food for their animals. There are many reasons why people seek alternative pet foods, which include doubting the nutritional value of the ingredients used in commercial diets, avoiding chemicals or ingredients they do not understand, having concerns about the effects of industrial processing, wanting to strictly control ingredients they believe to be good or bad, believing that cooking strengthens their bonds with their pets, wishing to offer a variety of foods or believing their dog dislikes commercial foods.

Establishing a homemade diet is not a simple task. It requires a complete understanding of the animal's nutritional requirements, the chemical composition of all ingredients, the formulation process and how pathophysiologic processes alter the animal's nutritional requirements^(^^1^^)^. The selection of appropriate ingredients should be based on their nutritional content, availability, palatability, and cost, as well as the owner's beliefs.

In a survey with dog and cat owners in the USA and Australia^(^^2^^)^, less than one-third of animals being fed homemade diets received a recipe balanced by a trained professional. In another study, the nutritional adequacy of forty-nine maintenance and thirty-six growing homemade diets for dogs was evaluated^(^^3^^)^. The majority of the diets (86 %) contained inadequate minerals, 55 % contained inadequate protein and 62 % contained inadequate vitamins. Animals can develop diverse disease conditions when they consume unbalanced diets, including osteodystrophies, pansteatitis, skin diseases, anaemia, hypoproteinemia, cardiopathies, nephropathies, immune deficiencies and many others. Among these diseases, osteodystrophies seem to be the most commonly reported for dogs fed homemade diets^(^^4^^–^^7^^)^, but epidemiological studies to determine the most prevalent nutritional diseases are not available. However, clinical signs associated with nutritional deficiencies or excesses may take a long time (months to years) to develop, and owners may mistake the lack of pathognomonic signs as evidence that the diet is appropriate.

The nutritional adequacy of homemade diets also depends on the owner's ability to strictly follow the recommended recipe. The person preparing the food may choose to remove, replace or change the amount of an ingredient for several reasons, including limited availability, high cost, personal beliefs or poor consumption by the dog. The owner, however, is not always able to evaluate the effect of these changes on the final nutritional composition of the food. Therefore, instructions about the diet preparation and storage, the serving sizes and frequencies, the importance and purpose of each ingredient and the specific amounts to use for each ingredient should be clearly explained to owners.

Despite these difficulties, properly formulated and prepared homemade diets may be nutritionally adequate and provide a useful tool for the clinical treatment of diseased animals^(^^8^^)^. Homemade diets may be used during the transition period after an animal is very sick and anorectic to increase its nutrient intake, to avoid inducing an aversion to commercial diets, in situations where there are no appropriate commercial diets^(^^9^^)^, when patients are diagnosed with more than one disease simultaneously, if the owner prefers homemade foods, or when a food hypersensitivity needs to be diagnosed^(^^10^^)^.

The nutritional adequacy of a homemade diet recipe is largely dependent on the nutritionist who formulated it. Although several publications showed inadequacies in recipes^(^^2^^,^^3^^)^, this could be corrected by properly training the professional. However, owner comprehension and adherence to the prescribed recipe is equally important because an adequate recipe can be made inadequate if the owner changes the ingredient composition. Unfortunately, data on how owners understand, translate and use the information provided by the nutritionist are limited. Therefore the present study evaluated how dog owners use and adhere to homemade diets prescribed by veterinary nutritionists, as well as the owners’ perceptions of the adequacy of the homemade diets for the nutritional support of their animals.

## Experimental methods

Fifty-nine owners who fed their dogs homemade diets prescribed by the Clinical Nutrition Service, Teaching Veterinary Hospital of the College of Agrarian and Veterinarian Sciences, São Paulo State University (UNESP) between March 2008 and January 2010 were selected for the present study. The animals underwent complete physical evaluations, anamnesis and a detailed nutritional assessment. The nutritional assessment included body weight, body condition score using a nine-point scale, skin and coat evaluation, muscular mass assessment, and complete dietary history and is consistent with the recently published guidelines for the nutritional evaluation of companion animals^(^^11^^)^.

Using the results of the nutritional assessment, clinical condition and diagnosis for each dog, a nutritionally complete and balanced homemade diet was prescribed. The ingredients used in the recipes included cooked rice, potato, beef, chicken, bovine or chicken liver, carrots, green beans, fish oil supplements, salt, soyabean oil, dicalcium phosphate, calcium carbonate and dried yeast, as well as commercially available vitamin, mineral and amino acid supplements to fulfil minor nutrient requirements. Not all ingredients were used in all diets. The nutritional composition of the diets met the recommendations by the National Research Council for dog maintenance diets^(^^12^^)^, and the diets were formulated using commercially available food formulation software. All owners received a written recipe that included the daily amounts of each one of the prescribed ingredients. The veterinary nutritionist carefully explained to owners the importance of following the recipe, the reasons for not changing the type or amount of each ingredient, the nutritional importance of each ingredient used, and details on how to prepare and feed the diet.

The survey was conducted over the telephone using a standardised questionnaire. Only owners who were using the homemade diet for at least 6 months were included. Owners were invited to answer questions by first reading all possible answers and then selecting the one that best indicated their opinion. The questionnaire included questions related to the owners’ difficulties in preparing the food, their adherence to the prescription, their subjective evaluation of the faecal and hair quality of their dogs and their perception about their dogs’ acceptance of the food and its effectiveness (Table 1). The results from the survey were evaluated using descriptive statistics, and the frequency of answers was expressed as a percentage.

**Table 1.** Questionnaire applied to owners of dogs fed homemade diets for at least 6 months included in the study

Questions	Possible answers
1. When you received the recipe, you thought:	a) it is easy to prepare
	b) you did not worry about it
	c) it should be difficult to prepare
2. Who prepares the diet?	a) you
	b) another person
3. During the preparation of the meal, the person who prepared it thought:	a) it was easy to prepare
	b) did not worry about it
	c) it was difficult to prepare
	What were the main difficulties?
4. Did you make any modification in the recipe?	a) no
	b) yes. Which one?
5. During the preparation of the diet, did you:	a) cooked all the ingredients
	b) cooked some ingredients
	Which ingredients did you supply uncooked?
6. How do you measure the amount provided of each ingredient?	a) I used a domestic scale
	b) I measured with a kitchen utensil (glass, cup)
	c) I used the guide given by the nutritional service
	d) I defined by myself how much to provide
7. Regarding the amount of ingredients, how did you proceed? (the question was repeated to each ingredient of the formula)	a) I put exactly the established amount
	b) I put more or less (approximately) the established amount
	c) I thought it was not enough, so I put more than the established amount
	d) I thought it was too much, so I put less than the established amount
8. Who feeds the dog?	a) you
	b) another person
9. The person who feeds the dog, thinks that	a) the dog likes the food
	b) the dog eats, but does not like it very much
	c) the dog does not like the food
10. About the ingredients	a) the dog eats everything
	b) the dog does not like some, and rejects it
	c) the dog does not like some, but end up eating because all the ingredients are mixed together
	Which are the ingredients the dog does not like?
11. Regarding the total amount of food provided, how do you proceed? (the question was repeated to each ingredient of the formula)	a) I provide exactly what is in the recipe
	b) I provide more or less (approximately) what is in the recipe
	c) I thought it was not enough, so I offered more than the established amount
	d) I thought it was too much, so I put less than the established amount
12. How do you measure how much the animal eats?	a) I use a measurer
	b) I weigh how much to provide
	c) I have a notion and provide how much the dog eats
13. What do you do when there is some leftover in the bowl?	a) I think it is normal, and do nothing
	b) Next meal I give less food
	c) I change the recipe adding what the dog likes the most to stimulate consumption
	d) There is no leftover
14. The faeces of your dog are	a) good
	b) a little soft
	c) very soft
	d) dried
15. Comparing with when the dog ate commercial food, how do you think the faeces are now	a) the same
	b) worse
	c) better
	d) never ate commercial food, always ate homemade food
16. Did you notice any modification in the coat	a) no, it is the same
	b) yes, it became worse
	c) yes, it became better
17. Do you think the homemade diet was	a) effective
	b) not effective
	c) indifferent (comparable with the previous diet)

## Results

Among the fifty-nine owners, twelve of fifty five (21·8 %) did not adhere to the prescribed diet and did not answer the questionnaire. Among them, seven of twelve (58·3 %) reported that their dogs preferred commercial food, and five reported that their dogs died due to the severity of their disease. For the forty-six owners that completed the questionnaire, forty three of forty six (93·5 %) used homemade diets because their dogs had some disease or had hyporexia, whereas only three of forty six (6·5 %) owners chose to use a homemade diet for their healthy dogs. Thirty seven of forty six (80·4 %) of the evaluated dogs were fed with a commercial diet before receiving the homemade diet prescription, seven of forty six (15·2 %) were previously fed a homemade diet without any regard for its nutritional balance and two of forty six (4·3 %) were previously fed a blend of commercial and homemade diets.

A number of diseases prompted the use of homemade diets, including ten of forty-three (23·2 %) patients with gastroenteric diseases, five of forty three (11·6 %) with some types of cancer, three of forty three (6·9 %) with heart disease, three of forty three (6·9 %) with infectious diseases, three of forty three (6·9 %) with kidney disease, three of forty three (6·9 %) with perineal hernias, three of forty three (6·9 %) with endocrine diseases, two of forty three (4·6 %) with obesity, two of forty three (4·6 %) with osteoarticular disease, two of forty three (4·6 %) with urolithiasis, two of forty three (4·6 %) with haemoparasitosis, one of forty three (2·3 %) with pseudocyesis, one of forty three (2·3 %) with liver disease, one of forty three (2·3 %) with pyoderma, one of forty three (2·3 %) with periodontal disease and one of forty three (2·3 %) with pancreatic disease.

The majority of the owners (forty one of forty six, 89·1 %) were responsible for preparing the food, whereas only five of forty-six (10·9 %) owners delegated this function to another person. Thirty-five of forty-six (76·1 %) owners reported that the diet was easy to prepare, whereas nine of forty six (19·6 %) believed that its preparation was difficult. Among the difficulties listed, owners reported that it was laborious to prepare the food (three of nine, 33·3 %), took a significant amount of time to cook (two of nine, 22·2 %), or was difficult to weigh or define the appropriate amount of ingredients (two of nine, 22·2 %); two owners did not indicate why they found the diet difficult to prepare (two of nine, 22·2 %).

All owners prepared the food by cooking all the ingredients according to the recommendations. Fourteen of forty-six (30·4 %) owners admitted to modifying the recipe by increasing, reducing, changing or eliminating some ingredients. To measure the appropriate amount of each ingredient, only seven of forty-six (15·2 %) owners had a domestic food scale and weighed the ingredients, whereas twenty-two of forty-six (47·8 %) owners used the volume guide provided in the prescription to estimate the amounts and seventeen of forty six (36·9 %) empirically defined their measurements and had no specific information on the amount they provided for each ingredient. When asked specifically about each ingredient, only twenty-three of forty-six (50·0 %) owners reported that they add the exact amount of meat, starch or vegetables, whereas the other half of the owners added more or less of these ingredients according to their beliefs. Only twelve of forty-six (26·1 %) owners used the prescribed amounts of soyabean oil or salt. More than 50 % of the owners specifically restricted or even omitted the soyabean oil and salt from their dogs’ diets. With regard to vitamin, amino acid and mineral supplementation, thirty of forty-six (65·2 %) owners reported using the exact prescribed amount, whereas thirteen of forty six (28·3 %) omitted these supplements from the diets and three of forty six (6·5 %) fed empirical amounts.

Owners of thirty-five of forty-six (76·1 %) dogs believed their animals really liked the homemade food, whereas six of forty-six (13·0 %) owners stated that their dog did not really like the food but ate it anyway and five of forty-six (10·9 %) owners reported that their dog did not like the food. Twenty dogs (twenty of forty-six, 43·5 %) consumed all their food, without leftovers. For those dogs which did not eat all the provided food, fifteen of forty-six (32·6 %) owners considered this normal and did not take any action, ten of forty six (21·7 %) changed the recipe by adding some food that the animal liked better to stimulate their consumption, and one of forty six (2·2 %) gave less food in the next meal. With regard to specific ingredients, thirty-three of forty-six (71·7 %) dogs ate all the provided ingredients, eight of forty-six (17·4 %) dogs did not like a specific ingredient and rejected it, and five of forty-six (10·9 %) dogs did not like a specific ingredient but ate it anyway because all the ingredients were mixed together. Vegetables (seven of ten, 70·0 %), liver (two of ten, 20·0 %) and mineral and vitamin supplements (one of ten, 10·0 %) were the ingredients most often disliked by the dogs.

Thirty-nine of forty-six (84·8 %) interviewed owners reported that their dogs’ faeces were well formed, six of forty-six (13·0 %) reported that the faeces were a little soft and one of forty-six (2·3 %) reported very soft faeces. For thirty-two of forty-six (69·6 %) owners, the faeces of their dogs were the same as when they ate commercial food. Forty-two of forty-six (91·3 %) owners did not notice a change in their dogs’ hair coat appearance after introducing the homemade diet, but two of forty-six (4·3 %) believed that their dogs’ hair coat appearance was worse and two of forty six (4·3 %) believed that it was better.

## Discussion

The present study gathered information about the use of homemade diets by dog owners and their opinions about using these diets. Most studies of homemade diets have evaluated the adequacy of the prescribed recipe, but in the present study, the adherence and overall impression of the owner was evaluated. The preparation of homemade diets may give owners the feeling of greater involvement with their pets^(^^10^^,^^13^^)^, and anecdotal information suggests that this practice is increasing. For 80 % of the owners in the present study, the time and labour required to prepare the food is not a problem, and they are happy to do it. Additionally, the adherence to food preparation and cooking was 100 %, which differs from studies in other countries where the consumption of raw meat and bones is high^(^^2^^)^ and associated with microbiological health risks^(^^14^^–^^16^^)^. However, this information cannot be generalised because only those who previously agreed to use homemade food were interviewed, which does not include the many people who do not wish to use homemade diets.

It was especially difficult to assess the nutritional balance of the diets that were being fed. Although all recipes were nutritionally complete and balanced and designed for the patient's health condition, as prescribed by the nutritionist, 30·4 % owners admitted that they had changed the recipe, 40 % did not adequately control the amount of provided ingredients, 73·9 % did not use the recommended amounts of soyabean oil and salt, and 28·3 % did not use the vitamin, mineral or amino acid supplements. This makes it impossible to estimate the actual nutritional composition of the diets that were incorrectly administered. Alterations in sodium (salt), fatty acids and energy (vegetable oil), fibre (vegetables) and amino acid, mineral and vitamin supplementation were expected, making the nutritional composition of the diet unpredictable. Meat was often used to stimulate food consumption, most probably due to the belief that a dog's diet must always contain large amounts of protein, potentially limiting the intake of other ingredients and inducing nutritional imbalances.

Inadequate control of the proportion of the ingredients and unauthorised substitutions are common mistakes made by owners who supply homemade diets^(^^17^^)^. The reduced supply of vegetables, which are usually the less acceptable ingredients to dogs, reduces fibre consumption. This may explain, in part, the occurrence of soft faeces in some animals and might increase the energy density of the diet, predisposing the dogs to excess energy intake. One interesting observation was the owners’ preoccupation with restricting salt and oil, exemplifying how human subjects transfer their nutritional concerns to their dogs. Hypertriglyceridaemia and hypercholesterolaemia are uncommon in dogs, and when they occur, they are mainly related to endocrine diseases and breed genetics, rather than to diet^(^^18^^,^^19^^)^. Excess sodium is not a cause of hypertension in dogs, which are tolerant to ingesting high levels of this macroelement^(^^20^^)^.

Almost one-third of the owners did not supply any vitamin or mineral supplement. In these cases, multiple nutritional deficiencies are expected, especially when the calcium sources are not fed. The occurrence of nutritional secondary hyperparathyroidism in dogs and cats receiving homemade diets without supplementation is well established^(^^6^^,^^7^^)^. The owners’ reasons for not adding these supplements included the inconvenience in obtaining them, their high costs, lack of acceptance by the animal and a lack of understanding of its importance in the diet. In addition, owners did not always understand that inadvertent substitutions of supplements by altering the brand or type could significantly alter the nutritional balance of the diet.

### Conclusions

Although homemade diets can be a useful tool in the nutritional management of healthy dogs and animals with a variety of diseases, many owners do not correctly use this type of food. Lack of adequately controlling the ingredient proportions and alterations in the ingredients used may change the nutritional composition of the diet, exposing the animals to nutritional diseases. The veterinary nutritionist needs to consider the owner's commitment to closely following the recipe and their comprehension of nutritional processes before deciding to recommend this type of diet for their patients.
